# 

**Published:** 1897-06

**Authors:** 


### MARRIAGES.

Dr. G. Howard Davison, of Altamount Stock Farm, Millbrook, N. Y., was married Wednesday, April 25th, at St. Bartholomew's Church, New York City, to Miss Marion M. Cheseborough, of the latter city.

### PERSONAL.

Dr. G. A. Johnson, of Sioux City, Iowa, and Dr. R. P. Steddom, of Galesburg, Ill., have been stationed at Kansas City, Mo., as inspectors under the Bureau of Animal Industry.

Dr. T. A. Bray has been transferred to the quarantine department.

Dr. Wm. C. Barth, inspector under the Bureau of Animal Industry, has been transferred to Wichita, Kansas.

Dr. W. Heck, Keokuk, Iowa, inspector under the Bureau of Animal Industry, has been ordered to report for duty at Kansas City, Mo.

Dr. Hugh T. Doris, of Pittsburg, is defendant in a $10,000 damage-suit.

Dr. R. W. Carter, of Jobstown, N. J., is manager of the famous Rancocas stud-farm at that place.

Veterinarian W. C. McMakin, of Raleigh, N. C., has taken sufficient interest in good roads to have received at the hands of his people the position of supervisor of roads for that district.

Drs. R. H. Harrison, of Atchison, Kas., and L. E. Day, of Kansas City, Mo., were before the civil service examiners in February last for the position of meat-inspectors under the Bureau of Animal Industry.

Dr. A. S. Wheeler, of New Orleans, has located permanently at the Biltmore farms of Mr. George Vanderbilt, at Biltmore, N. C.

Veterinarian Robert Ward, of Baltimore, Md., has succeeded in having the Maryland School for Horseshoeing incorporated.

Dr. H. D. Hanson, of New York, purchased the trotting gelding Harry G., record 2.19½, at the April Fasig sale.

Dr. F. Rawson, of Kalamazoo, Mich., has removed to Lawton, in the same State.

Veterinarian L. Hugo Reed, of Guelph, Ontario, was the winner of the first prize for the best pair of carriage-horses shown at the Guelph horse-show.

Dr. J. Rein Keelor, of Harleysville, Pa., was recently chosen a director of the Lansdale Driving Park Association.

Dr. George Hilton, of Winnipeg, Man., has finally located at Portage la Prairie, Man.

Veterinarians James B. Rayner, B. M. Underhill, W. P. Phipps, B. F. Eshelman, and Warren T. Edwards were interested visitors at the May Stud Horse Show at West Chester.

F. L. Forrest, V. S., formerly of Defiance, Ohio, has enlisted as a private in the 19th Infantry, U. S. A.

Dr. Marsh L. Brackbill, Reading, Pa., is making a trip through Europe, having sailed with a consignment of horses.

